# SNORAP: A Device for the Correction of Impaired Sleep Health by Using Tactile Stimulation for Individuals with Mild and Moderate Sleep Disordered Breathing

**DOI:** 10.3390/s17092006

**Published:** 2017-09-01

**Authors:** Mete Yağanoğlu, Murat Kayabekir, Cemal Köse

**Affiliations:** 1 Department of Computer Engineering, Faculty of Engineering, Ataturk University, Erzurum 25240, Turkey; yaganoglu@atauni.edu.tr; 2 Regional Training and Research Hospital, Sleep Disorders Center, Electrophysiology Laboratory, Erzurum 25240, Turkey; 3 Department of Computer Engineering, Faculty of Engineering, Karadeniz Technical University, Trabzon 61080, Turkey; ckose@ktu.edu.tr

**Keywords:** sleep physiology, snore, sleep apnea, audio fingerprint, heart rate sensor, wearable sensor, sleep quality

## Abstract

Sleep physiology and sleep hygiene play significant roles in maintaining the daily lives of individuals given that sleep is an important physiological need to protect the functions of the human brain. Sleep disordered breathing (SDB) is an important disease that disturbs this need. Snoring and Obstructive Sleep Apnea Syndrome (OSAS) are clinical conditions that affect all body organs and systems that intermittently, repeatedly, with at least 10 s or more breathing stops that decrease throughout the night and disturb sleep integrity. The aim of this study was to produce a new device for the treatment of patients especially with position and rapid eye movement (REM)-dependent mild and moderate OSAS. For this purpose, the main components of the device (the microphone (snore sensor), the heart rate sensor, and the vibration motor, which we named SNORAP) were applied to five volunteer patients (male, mean age: 33.2, body mass index mean: 29.3). After receiving the sound in real time with the microphone, the snoring sound was detected by using the Audio Fingerprint method with a success rate of 98.9%. According to the results obtained, the severity and the number of the snoring of the patients using SNORAP were found to be significantly lower than in the experimental conditions in the apnea hypopnea index (AHI), apnea index, hypopnea index, in supine position’s AHI, and REM position’s AHI before using SNORAP (Paired Sample Test, *p* < 0.05). REM sleep duration and nocturnal oxygen saturation were significantly higher when compared to the group not using the SNORAP (Paired Sample Test, *p* < 0.05).

## 1. Introduction

Sleep physiology and sleep health play very important roles in maintaining the daily lives of individuals. Sleep is an important physiological need to protect the functions of the human brain. While sleeping is an activity that must be performed at night, the duration and depth of sleep, and the number of awakening episodes at night all directly affect sleep quality. High sleep quality leads to more accurate functioning of healthy individuals’ brains and a high quality of daytime activities.

In general, ventilation decreases during sleep, fluctuates in the tidal volume and as periodic respiration starts, blood pressure and heart rate decrease [1]. Two different sleep phases have different effects on respiratory and cardiovascular systems: (a) Non-REM (NREM, calm, synchronized sleep, deep-wave sleep); and (b) REM (moving, desynchronized, paradoxical sleep) [2]. Upper airway resistance, approximately doubles during NREM sleep; and no significant change is expected in the lower airway resistance. During sleep, the voluntary control of breathing and the stimulation of alertness are disabled, and breathing control is provided by metabolic stimuli. Respiration becomes irregular in the REM period: hypotonia develops in skeletal muscles and the sensitivity of the respiratory center to the nearby carbon dioxide is decreased. Respiratory irregularity is evident in the period of eye movements [3,4]. In NREM sleep, heart rate and blood pressure fluctuations decrease and are lower than wakefulness. During the REM period of sleep, blood pressure rises and becomes irregular, but maintains levels below wakefulness levels [5].

There are many biological, psychological, and social factors that disrupt sleep physiology. Respiration disorders while sleeping are a very important disease group that disrupts sleep physiology in the area of sleep medicine. In particular, Snoring and Sleep Apnea Syndrome is a serious threat to the health of people and society, and most individuals are not concerned with snoring during sleep. However, individuals with Sleep-Disordered Breathing cannot get enough oxygen during nighttime sleep, and vital organs such as the brain and heart are not sufficiently oxygenated. Snoring is a stage that occurs during sleep, usually due to the vibration of the tissues in the upper airway, generally on inspiration, more rarely on exhalation and sometimes in both phases of respiration [6,7].

Sleep Apnea Syndrome is a clinical condition that affects all body organs and systems that interfere with sleep quality throughout the night, with recurrent intervals during the night that last at least 10 s or more and continue with breathing stops and reductions. It is typed based on anatomical location: (a) Obstructive Type; (b) Central Type; and (c) Mixed Type; according to size of affected by disease: (a) Mild Degree; (b) Moderate Degree; and (c) Severe Degree; and according to sleeping periods and position it varies as: (a) REM Dependent; and (b) Position Dependent. REM sleep is a situation when all skeletal muscles in the body except the diaphragm incur atonia while active, and episodic REM occur at electroencephalography (EEG). Therefore it is referred to as paradoxical sleep. Skeletal muscles providing upper airway tone in patients with OSAS incur atonia more frequently; thus, snoring and apnea attacks increase.

Polysomnography (PSG) is the gold standard method for the diagnosis of OSAS. The assessment of the sleep stages and respiratory events in sleep was performed as per the guidelines of the American Academy of Sleep Medicine. Apnea was determined as an oro-nasal airflow cut for at least 10 s. Hypopnea was defined as a 3% decrease in oxygen saturation with a reduction of at least 50% in oro-nasal airflow or accompanying arousal. Arousal was defined as waking up while sleeping or passing to a more superficial sleeping environment. Characteristic PSG findings of OSAS can be listed as follows: (a) an increase in the duration of superficial sleep, and a decrease in that of deep sleep and REM is observed; (b) The recurrence of apnea and hypopnea recur frequently; (c) Oxygen desaturations recur frequently (d) REM sleep increases the frequency and duration of apnea, and the degree and duration of oxygen desaturation. Supine sleeping position also makes contribution to this increase; (e) Paradoxal chest and abdominal movements are typically seen during apnea; (f) Heart rate usually slows down during apnea and accelerates after apnea; arrhythmias can be observed during this period; (g) Irregular snoring that frequently recurs and interrupted by apnea is heard in respiratory voice recording. The degree of disease is determined by AHI (apnea hypopnea index; obtained by dividing the total number of apnea and hypopnea seen in sleep by the time of sleep in hours) value detected according to PSG result. If this index is greater than 5, sleep apnea syndrome can be a matter. But, the clinically important value is 15 and above. OSAS classification, based on the AHI values, are given in Table 1 [8,9].

Among these disease groups, Continuous Positive Air Pressure (CPAP) therapy appears as the gold standard treatment method, especially in moderate and severe degree OSAS. However, both REM-dependent and position-dependent patients with simple snoring, moderate, and mild OSAS cannot be proposed with CPAP therapies and cannot be treated completely. However, every grade of the disease leads to different symptoms that are specific to the person.

Daytime complaints of patients can be listed as “daytime sleepiness (when driving, doing important work, reading a book), attention and concentration disorders, daydreaming and headache in the morning, and psychological problems (such as anxiety, depression, emotional disturbances, etc.)”; and nighttime symptoms are “snoring, discomfort given to the bed partner and other members of the house due to snoring, chest pain, drowsiness feeling in sleep, poor sleep quality due to short vigilance, night headache”. All of these symptoms, unfortunately, remain in the patient group, listed in Table 1, which cannot be fully treated. Our hypothesis was that using the device we developed for the treatment purposes will be good for the disease groups shown in Table 2.

The purpose of this study was to develop a new device for the treatment of a special group of patients whose sleep and wakefulness health has been impaired by sleep disorder.

## 2. Related Works

Recently, studies to find audio fingerprints have become popular, with even large corporate institutions developing algorithms in this area. The most well-known audio fingerprinting algorithm is Shazam [10], which is based on local audio fingerprints. With Shazam, people can find songs they seek using smartphones as the application uses the peaks observed in the spectrogram of the audio signal as local feature points of the song. Property descriptors are then generated from the attributes of these pairs of points, and a compact fingerprint time difference forms for each pair of frequencies on each pair and for each pair [11].

Spectrograms, signal and image processing methods are often used in audio fingerprinting algorithms. An algorithm based on spectrogram extraction of general fingerprint based audio signal has been presented by Haitsma and Kalker [12]. Waveprint, a wave-based audio fingerprint algorithm has also been suggested by Baluja et al. [13] and used recently by the Google voice search system. The Waveprint key algorithm is based on wavelets, which is robust with respect to codec or a bit rate change. Zhu et al. [14] introduced a constant scale feature transform-based algorithm. Chunk and Ko [15] proposed a new method that could reduce the number of fingerprints by using Gaussian difference, which is used for feature extraction during image signal processing. Cano et al. [16] defined a voice recognition system with various voice fingerprint features, and Rein and Reisslein [17] proposed a method for the use of audio fingerprints to identify a classical music composition that could not be identified through the use of perpendicular wave dispersion vectors and neural networks. In the study by Ellis et al. [18], a spectrogram was created and the starting points of the points were found using this spectrogram. Fingerprints have been produced by using time differences between these points.

Various studies have been carried out to measure sleep apnea diagnosis and sleep quality. In their study, Lazaro et al. used signal processing to predict sleep apnea and respiration rate [19], while Nam et al. demonstrated a new monitoring system to measure sleep quality [20]. In their study, Adnane et al. presented a new method for detecting apnea periods by using signal processing packages [21]. Recently, different techniques for sleep apnea monitoring have been widely developed [22]. Additionally, Nam et al. suggested the estimation of the correct respiration rate from a smartphone by using breath sound recordings from the nose: the proposed method detects the nasal airflow by using an interior smartphone microphone or a headphone microphone placed under the nose [23]. Nguyen et al. used heart rate complexity measures to classify OSA events [24]. Le et al. proposed a wireless placeable model in their studies to anticipate sleep apnea attacks in advance. They developed an approach to provide an early warning of 1–3 m for the oncoming sleep apnea area .They calculated the accuracy of offline OSA classification as 88%,that of predict it 1 m ahead as 83%, that of predict it 3 m ahead as 77% [25]. Bukkapatnam et al. took out a patent on a wireless wearable sleep apnea treatment system. This patent includes a wearable sensor vest for use in the treatment of sleep apnea. It also includes an EKG monitor and a wireless signal receiver card, being in touch with EKG monitor and computer, and allows for electricity reading from EKG monitor to computer, and a patient’s stimulus controlled in order to receive the patient signal [26]. Afrin et al. developed a home sleep tester, clinically almost equivalent to PSG system, low cost and easy to use and buried in an electronical sleep pillow [27].

## 3. Proposed Hardware Platform

**Figure 1:**
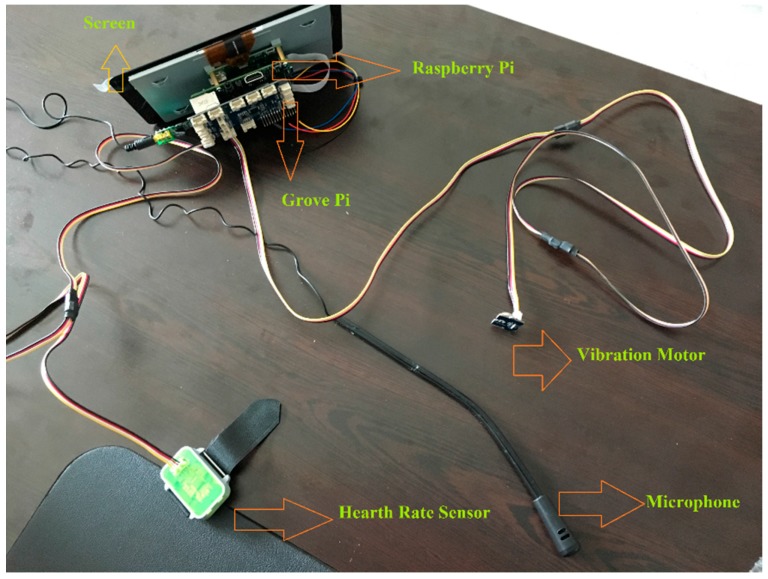
SNORAP: a prototype device consisting of 6 parts.

The SNORAP device was designed to prevent snoring and apnea (stopping breath for at least 10 s in sleep), one of the most common and important causes of disturbance to an individuals’ sleep health. Our device operated as real time as a wearable device. As shown in Figure 1, our device consisted of six parts. The display uses the Display Serial Interface (DSI) connector on Raspberry Pi and shows the results of our application visually so that on-screen heartbeats and snoring sounds can be displayed instantly.

Raspberry Pi is a mini-computer the size of a credit card. The technical specifications of the Raspberry Pi include: 1.2 GHz 4-core 64-bit quad-core ARMV8 processor, 1 GB RAM memory, Bluetooth 4.1, 40 GPIO, four USB 2 ports, full size HDMI port, CSI camera port for connecting the Raspberry Pi camera, DSI display port for connecting the Raspberry Pi touch screen display, micro SD socket. Grove is a shield card that allows Raspberry Pi to connect to Grove sensors. There are 15 Grove sensor connections on the card. A grove sensor card has 15 4-pin Grove sensor connections on it. Grove Pi + is fixed on Raspberry Pi without the need of any other connection. The communication between them is carried out over I2C interface. All Grove modules are connected to the Grove Pi + card via the universal 4-pin connector cable. Grove modules with analog or digital output, are connected to an ATmega 328 micro controller (Microchip Technology, Chandler, AZ, USA) on the grove Pi + card. The micro controller acts as an interpreter between Raspberry Pi and Grove sensors. It issues and receives commands and runs commands that are issued by the Raspberry Pi. The screen produced by the 7-inch Raspberry Pi official manufacturer uses the DSI connector on Raspberry Pi, and thus, it does not occupy the HDMI port and GPIO pins. Its features: RGB 800 × 480 resolution, 60 fps support, 24-bit color depth, touch support up to 10 points by means of FT5406 touch-operated controller, 70° visual angle [28,29].

The heart rate sensor module can measure the variation in human blood movement in the veins thanks to optical technology. It has CMOS with high performance and low power consumption, using PAH8001El-2G (PixArt Imaging Inc., Hsin-chu, Taiwan) in the sensor measuring heart beat via a finger clip. Since the heart rate sensor chip should have a high processing speed for the algorithm of heart rate data, STM32 (STMicroelectronics, Geneva, Switzerland) is integrated into this module. The heart rate sensor performance was calculated in real-time, and was found to have an average difference of 0.55 bpm compared to the PSG system. The microphone was used to receive and process voice in the environment, and the vibration motor sent a warning to the user to perceive touch. The vibration sensor is in the size of a coin with a DC motor. It will vibrate when its entrance is logic High, in other words, the vibration motor is activated when a snoring sound occurs. It produces a very low- decibel sound that will not awake the patient. The vibration sensor included in SNORAP can be set according to individual’s perception characteristics of tactile stimulus. Thus, patients who need more stimulation are sent stimuli by increasing the severity of the vibration. The snoring sound detection success was 98.9%. The 98.9% accuracy rate assigned for the SNORAP device, the study’s subject, corresponds to the snoring volume. Our device does not directly measure the duration or severity of apnea periods, but rather it indirectly reduces or eliminates OSA periods that occur after snoring periods.

**Figure 2:**
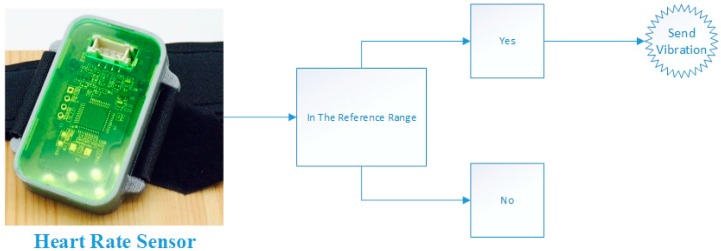
Sensor that detects heart rate changes.

As shown in Figure 2, if the heart rate sensor connected to the user (with a finger clip) was above or below the specified reference value (40–120/min), the user sent frequent vibrations. Here, our goal was to alert patients with a heart rhythm problem during sleep.

Particularly for patients with mild and moderate sleep apnea (position and REM dependent), the snoring and apnea prevention device is very well operated by the snoring intensity and frequency of the person. The intensity and frequency of the snoring voice also points to the apnea, which may actually occur, especially when the patient is in a supine position and in REM, increased snoring attacks are sensed and vibration stimuli are sent to the body. The person then changes position so that snoring is decreased and the apnea that may occur afterwards can be eliminated.

SNORAP is a real-time-operating device, which can be used for about 10 h during the night, and is easily placed on the patient’s arm. The device is suitable for long-time use, and its lifelong use is recommended for patients. The electrical stimulus (delay time) issuing time of the device after perceiving a snoring sound is approximately 3.4 s. The severity of the stimulus is adjusted depending on the perception threshold of the patient, to whom stimuli are issued by sending vibrations to the patient’s arm in three 3-s bursts with 1-s intervals. This process, which lasts for 11 s in total, disappears spontaneously when the snoring noise is removed. When snoring attacks occur again, the same function is once again activated.

## 4. Method

An audio fingerprint is a short summary of an audio file [30]. Recently, many voice search sites (especially Google) have used audio fingerprint technology to search for the same voice. Audio fingerprints are often used for similar audio files and for content-based rotation of the same.

**Figure 3:**
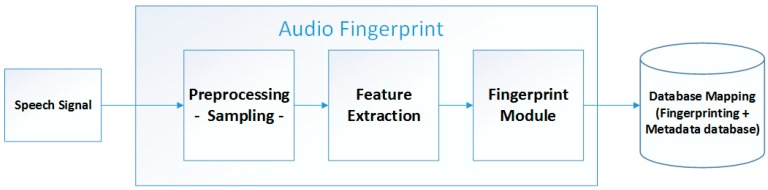
Sound/Speech Recognition System.

As shown in Figure 3, the Fingerprint detection module converts the perceptual characteristics of the sound recording into a solid short form (fingerprint). This module consists of three sub-modules: pre-processing, feature extraction, and fingerprinting modules. The pre-processing module provides the signal converting into ready-to-process format by performing some operations on the signal such as conversion from analog to digital, dropping single channel, and changing the sampling rate. The feature extraction module measures some predefined, distinctive values related to the signal. Measurements of a predetermined, distinctive value of the signal transformed into the frequency domain are made. A wide variety of methods can be used at this stage. Our aim was to reduce the dimension and increase the endurance against the distortion of the fence. The fingerprint modeling section also reveals the last fingerprint form of these measurement values. When a fingerprint is given, the fingerprint matching module compares this fingerprint with other fingerprints defined in the database and finds the best match.

In this study, the Audio Fingerprint method was used for snoring sound detection. One of the factors that determines the usability of a recognition system is so that it can effectively compare an unknown audio track with millions of known audio tracks. The Audio Fingerprint extracts the summary of the audio content and stores it in the database. In addition, the fingerprinting system must also be efficient in terms of calculation. Efficiency has a critical effect on both the calculation of fingerprints of unknown voices and in real practice, and even more so in the search for the best match in a large fingerprint database. Calculation costs are related to fingerprint size, search algorithm complexity, and fingerprint extraction complexity. The general approach is to create an index structure to reduce the number of distance accounts that will be made when a query is given. Many indexing methods group similar classes, ignore some classes, and search for the rest of the classes. The duration of an audio data database should be as short as possible, especially for real-time applications where it is important that the duration of the call is short. It is expected that call duration will not increase too much if the number of audio fingerprints in the database increases too much. In our real-time system, it took 3.4 s for our system to find out if there was no snoring sound or other sounds.

Audio fingerprint techniques aim to deliver successful results when content-based audio recognition is performed, even when audio signals are mildly or severely broken. In order for audio fingerprint to be successful, first, it is necessary to significantly reduce the size of the input audio signal. Second, resulting properties must be robust against possible degradation of the entrances. For example, if the songs playing on the radio are to be detected, the system must be robust against any non-linear distortions that most stations identify to the pre-broadcast signal. Third, the resulting features should be informative: for sound identification, different sound clips must be matched to distant features in some appropriate metrics. Finally, the calculation in the feature extraction process must be efficient [31].

**Figure 4:**
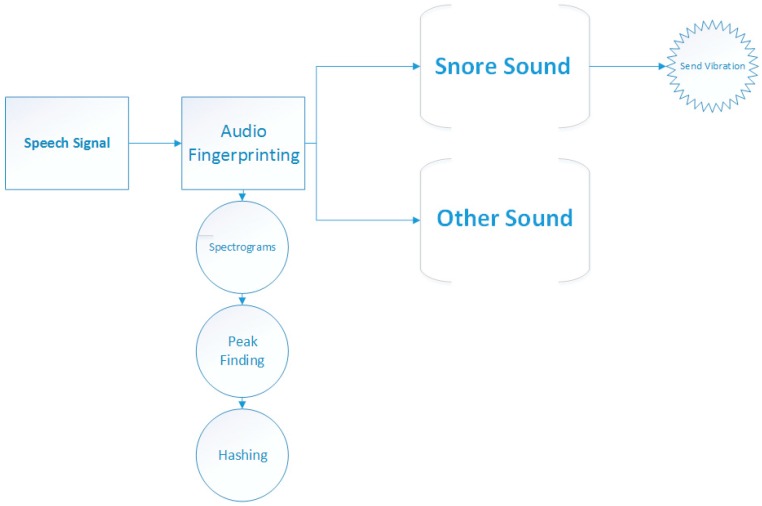
SNORAP Working Mechanism.

As shown in Figure 4, spectrographs were obtained especially from the audio data. Next, the peaks were found and a summarization fingerprint was generated. Then, our system determined whether this voice was a snoring voice or other sound. Our system detected a snoring voice at 98.9%. After the snoring sound was detected, it sends out vibrations to the person, so that extraction from snoring was provided and he was prevented from entering sleep apnea.

### 4.1. Preprocessing and Sampling

In the front processing section: first, the sound was converted into digital form and brought into a common, designated form. In the case of audio recording, the accepted rule is that human ears miss frequencies above 22,050 Hz. For this reason, there were 44,100 samples in the second as per the Nyquist-Shannon Sampling Theorem.

### 4.2. Spectrograms and Peak Finding

As the samples were a kind of signal, the Fast Fourier Transform was repeatedly used in small time windows from the samples of the sound to form the sound spectrogram. Peak points were preferred in this study given the high possibility of peak protection in cases of audio distortion. As they were highly resistant to noise and signal distortion, they used spectrogram peaks as their fingerprints. Fingerprints were created using the frequency values and time differences of the peaks found.

**Figure 5:**
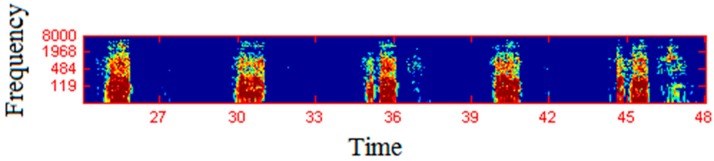
Spectrogram of the snoring sound.

Figure 5 shows the spectrogram of the snoring sound, which was a 2-dimensional array with a wave amplitude as the time and frequency function. The applied FFT showed the special frequency signal by providing a column at the end. Sufficient windowing was required to obtain a 2-dimensional array spectrogram. The spectrogram was used to describe the uniqueness of a voice. As various sounds cause noise in the outdoor and indoor environment, our goal was to catch the most discriminating fingerprint of the audio signal. First, we had to find the amplitude peak points from the spectrogram of the audio signal. The time corresponding to the amplitude of the largest amplitude from the neighborhood around the peak was frequency, which lowers the amplitudes of the other pairs around it and reduces this noise. Even if new peak points occur due to noise, the other peaks will not be affected much as the peaks are locally independent of each other. If peaks are deleted in a similar way, most of them will be preserved. The display of the summit points is called a constellation map.

It is a slow process to find the right audio records and starting point by using the peak points directly as they do not have enough dependency. Instead, fingerprints were obtained by pairing the peaks in the constellation map in binary combinations. The anchor point was selected and each anchor point had a target region. Each anchor point was sequentially paired with the peak points remaining in the target region.

### 4.3. Fingerprint Hashing

As there may be similar peaks when peaks are found, we found the appropriate trace for that sound by combining the peaks with fingerprints by using a hash function. The summarization function took an integer as input and converted it to another integer as output. By looking at the peaks of the spectrograms and the time differences between peak frequency combinations, we could create a summation to distinguish sounds. This study was developed based on Shazam’s method [10], where the fingerprint having more detailed entropy (in other words, containing more information), was formed by accounting for more than one peak. In this study, the SHA-1 summarization algorithm was used for summarization.

Some match-up was obtained with some audio tracks as a result of the search in the database. The distance account methods were used in order to express the matching amount with audio tracks. The audio tracks with the shortest distance are the most probable candidates. The audio features used in audio analysis can be basically divided into two groups: high-level features and low-level features [32]. The high-level features include the kinds of knowledge that an individual obtains while listening sound. The features such as timbre, melody, rhythm, pitch, harmony, structure, and lyrics can be included in this group. The high-level features can be used for snoring-sound detection. However, the sound events that we considered in further studies and the spectrogram peaks, more resistant to noises, were used as the fingerprint feature in this study. A time-frequency point in a spectrogram is the peak point if it has a higher energy level than all neighbors in a region around it.

### 4.4. Experimental Protocol

SNORAP is a wearable device designed for home use. The present study consists of two parts: The first stage was the design and construction of the SNORAP device, which lasted about 1 year. The second stage was an experimental process that was conducted within 2 months at a sleep disorders center, operated by a medical doctor who received education in sleep medicine, accredited according to international rules, and having received its ethical approvals. Collection of data from volunteer patients was done in real-time during their night sleeps. The SNORAP application has been used on diagnosed patients by means of PSG findings belonging to volunteers staying at the lab for the first night after 5 days. Thus, the situations of patient before and after the use of SNORAP have been easily compared. The data collection period for each volunteer is 7 days. The volunteers were examined by a medical doctor (MD, specialist physiologist) and their one-night tests were conducted in company with sleep medicine technicians. Before doing this study, the volunteer patients were trained in sleep medicine and examined by a medical doctor; the recorded PSG results were analyzed by the same physician in charge of the laboratory, and the diagnosis of OSAS was made in compliance with American Academy of Sleep Medicine (AASM) criteria.

The test protocol consisted of two steps. The first consisted of the PSG recording and diagnosis phase; the second consisted of the phases where PSG and SNORAP applications of diagnosed patients were performed together. SNORAP was easily compatible with the PSG sensors proposed by AASM. It was also observed that snoring attacks occurring during the night are detected by PSG sensors; in addition, the SNORAP device simultaneously and in real time detected the same snoring attacks. The decrease in and removal of snoring and apnea attacks when SNORAP perceives snoring and issues a tactile stimulus to the patient was clearly monitored with the PSG sensors recommended by AASM.

**Figure 6:**
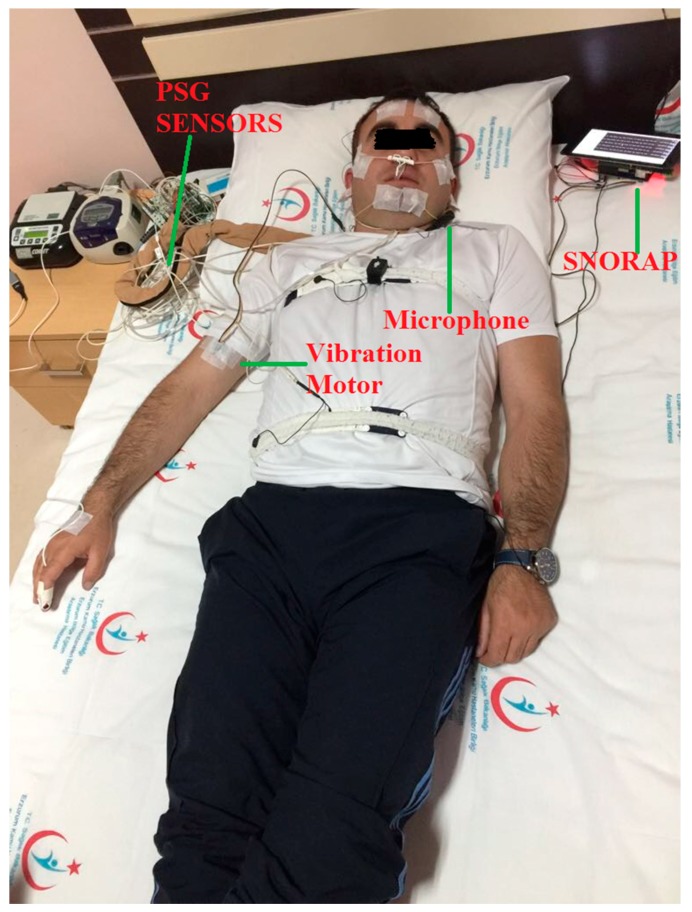
Polysomnography and SNORAP practice during a night in a sample patient.

The experimental procedure for the device was tested on a total of five volunteer patients with a respiration disorder during sleeping (male, mean age: 33.2, body mass index average: 29.3), and only five polysomnographies (standard method for determining sleep disturbances) were recorded by the five volunteers. PSG records were taken with the application of the prototype device shown in Figure 6.

PSG included six channels of EEG, two channels of electrooculography (EOG), one channel of submental muscle electromyography (EMG), two channel of EMG placed on both anterior tibial muscles, one channel of oro-nasal airflow cannula, one channel of oro-nasal thermal sensor, inductive plethysmography to demonstrate the two-channel chest and abdominal breathing effort, a channel of “body position” sensor, a channel of finger probe and pulsoximeter to measure arterial oxyhemoglobin saturation (SpO2), and simultaneous video recording.

## 5. Experimental Results

### 5.1. Snoring

**Figure 7:**
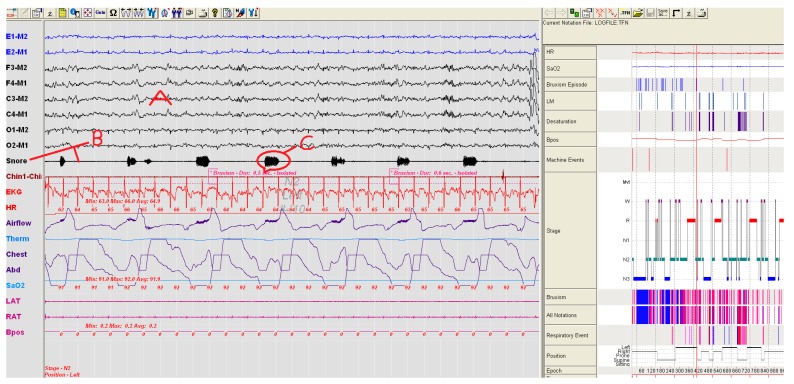
A patient sample not using SNORAP—high volume snoring sounds are observed in the snore line (A: 6-channel EEG, B: Snoring trace, C: high amplitude snoring packets).

**Figure 8:**
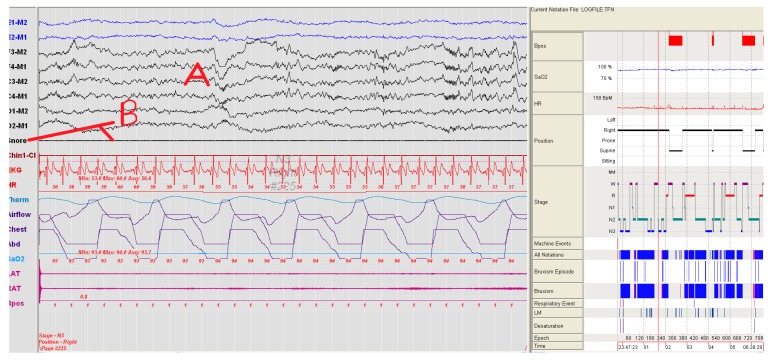
An image of the same patient in Figure 7, while sleeping by using SNORAP and the snoring is completely disappeared (A: 6-channel EEG, B: Snoring trace and it is seen that the high amplitude snoring packets are completely removed).

Using the PSG, the number and severity of snoring for each patient were compared. Snoring severity and number of the group using SNORAP were found to be statistically lower (Paired Sample Test, *p* < 0.05) than when compared to the group not using SNORAP. A volunteer patient from study groups in Figure 7 and Figure 8 was shown a sample PSG recording before and after using SNORAP. 6-channel EEG (A) and snoring trace (B) are given in Figure 7. The high amplitude snoring packets (C) are seen in the snoring trace in Figure 7. Figure 8 shows the PSG recording of the same volunteer patient when the SNORAP was applied to that patient. When his snoring trace in these conditions is reviewed, it is seen that the high amplitude snoring packets are completely removed. No wave of being awake was observed in his sleep EEG.

### 5.2. Apnea-Hypopnea Index

**Figure 9:**
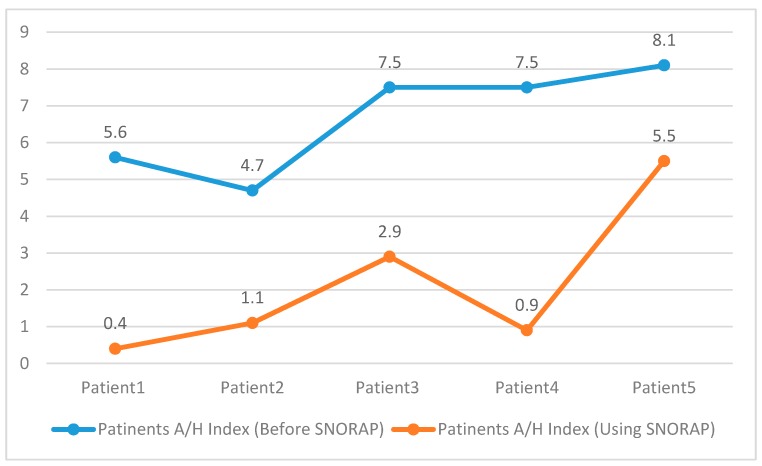
Patient’s Apnea/Hypopnea Index.

In the PSG analyses performed on each patient, the apnea-hypopnea index (AHI), apnea index, hypopnea index, AHI in REM and AHI in supine position were compared. As seen in Table 3, the values of SNORAP group were found to be low and statistically significant (Paired Sample Test, *p* < 0.05) when compared to the group not using SNORAP. As seen in Figure 9, the AHI value of patients using SNORAP seriously decreased.

### 5.3. Total Apnea/Hypopnea Count

**Figure 10:**
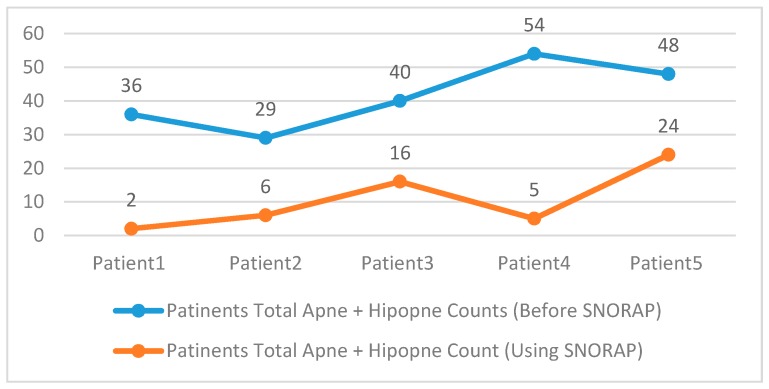
Patient’s Total Apnea + Hypopnea Counts.

As seen in Figure 10, the total apnea/hypopnea count of patients using SNORAP seriously decreased.

### 5.4. Rapid Eye Movement Sleep Time and Nocturnal Oxygen Saturation

**Figure 11:**
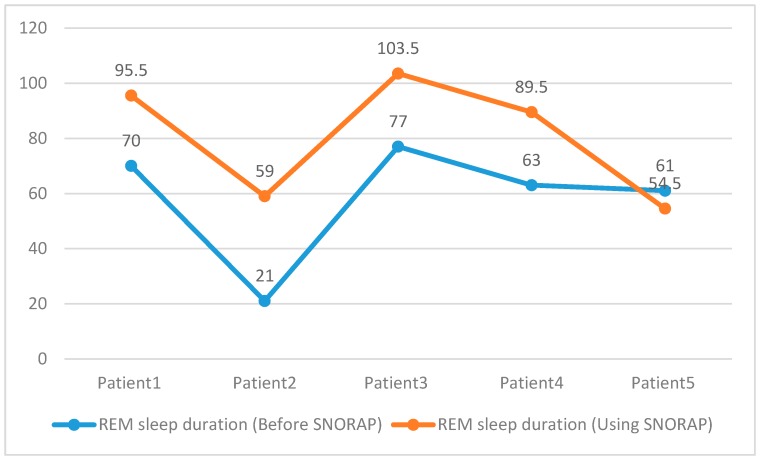
Rapid Eye Movement Sleep duration.

**Figure 12:**
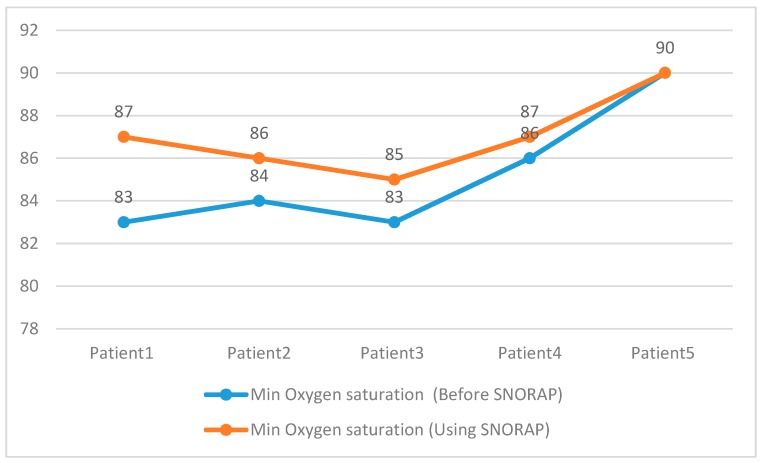
Min O_2_ Saturation.

When the results of PSG were examined, REM sleep duration and nocturnal oxygen saturation were compared (Figure 11 and Figure 12). Rear sleep duration and nocturnal oxygen saturation were found to be statistically significant (Paired Sample Test, *p* < 0.05) when compared to the group not using SNORAP.

## 6. Discussion

To improve the impaired sleeping health of people with mild and moderate SDB through the tactile stimulus system that we created, we first diagnosed the individual’s diseases as polysomnographical before setting up a five-person experimental group where the probable effects of the device (named as SNORAP) on the patients and the diseases detected. According to the obtained results, snoring severity and number of patients using SNORAP were found to be significantly lower than the experimental conditions before using SNORAP in the apnea-hypopnea index, apnea index, hypopnea index, supine position AHI and REM. The REM sleep duration of the group using SNORAP and nocturnal oxygen saturation were found to be significantly higher when compared with the group not using SNORAP.

### 6.1. Detection of Snoring Sound

The snoring sensor, which was the most important part of our device, was developed with the right methods and easily perceived the snoring sound. If you are working with a high sensitivity to snoring, your contribution to the treatment of sleep related respiration disorders is high.

In Ke et al. [33], computer vision techniques were used for voice recognition where the sound wave signal was converted into two-dimensional representation as time-frequency, and 33 Bark-frequency cepstral coefficients (BFCC) tape was used. In our study, the peak points were found after the time-frequency conversion and fingerprints were generated.

In their study, Baluja and Covell [13] used image processing methods to first create a spectrogram, before the Haar Wavelet was applied to the spectrogram images. As a result of this process, the wavelet coefficient of the number of pixels in the spectrogram picture appeared and the authors decided that only the most powerful t-wave would be sufficient. Ellis et al. [18] divided the spectrogram into eight bands where note starts and fingerprints were calculated for each band. In our study, after the spectrograms were generated, they were obtained by pairing the peaks, fingerprints, and peaks in the constellation map in binary combinations.

In the work of Burges et al. [31] a perceptually aggravated log spectrogram was used and the Distortion Discriminant Analysis (DDA) method was used to produce tolerance fingerprints for this spectrogram noise. In our study, the peak points were used to reduce noise. Several methods have been used in the study of snoring sound detection [34,35,36,37,38,39]. These studies, while trying to find the feature for voice in general, all tried to find the distinguishing feature of the voice and determined that the classifier and voice were snoring sounds.

In our study, the distinguishing features of snoring sounds were detected by taking fingerprints, thus at good classification success was ensured even in noisy situations. A number of methods have been developed to detect the vocalization of the soft tissues of the upper airway during sleep. As shown in Table 4, our method achieved better success than the other methods.

### 6.2. Snoring

Snoring is an important complaint seen in 44% of the middle-aged male population and 28% of the female population. It is a social problem that causes shame in society, and even affects marriages. The volume of the snoring can vary between patients and between nights in the same patient; it can be continuous or intermittent. Simple snoring is a condition that does not lead to regular sleep breaks; however, if awakened by a bed-mate, it causes sleep disturbances and insufficient sleep. Sleep apnea syndrome is the most common symptom. In males with snoring, the risk of developing OSAS within 10 years has risen [40]. In multivariate analysis, hypertension was predicted to be 1.4 times higher, myocardial infarction 1.34 times, and stroke 1.67 times more severe than the other variables, regardless of age, gender, body mass index, diabetes, education level, smoking and alcohol consumption [41]. The position with snoring and in the case with REM dependent, mild and moderate sleep apnea syndrome is mostly untreatable. In our study, the snoring and apnea attacks were stopped by using the SNORAP device, which had a very high sensitivity to the snoring sound, the prototype of which we designed, leading to a decrease in the severity and the number of snoring.

### 6.3. Apnea-Hypopnea Index

It is typical that snoring in OSAS patients is interrupted by frequent recurrent apneas. Patients often refuse to snore, although there is a common finding in almost all OSAS patients. Weight gain and alcohol intake are important predisposing factors [42]. AHI is the number of apnea and hypopnea occurring during sleep, divided by the number of days spent in sleep. This index determines the degree of sleep apnea syndrome. In the treatment of OSAS of a mild (AHI: 5–15) and moderate (AHI: 15–30) degree (especially for position-dependent type), it is advisable to classically put a hard tennis ball on the back of the nightgown and lie sideways. As a group disease, snoring and accompanying apnea attacks increase when the person stays in the supine position during sleep. The approaches recommended to the patients and position trainings were not performed by the patients or they remained insufficient. However, this situation, which is the subject of this study, was removed by means of the prototype device that we created. Patients come to the lateral position from the supine position, thanks to SNORAP, which attaches to the arms and sends tactile stimuli depending on snoring sound intensity. The apnea index, the hypopnea index, especially the AHI index at the supine position, were at the desired levels thanks to the device, which had a high patient compliance.

### 6.4. REM Sleep Time and Nocturnal Oxygen Saturation

Characteristic findings of OSAS as polysomnographical are the increase in superficial sleep and deep sleep, and decrease in REM duration. REM sleep increases the frequency and duration of apnea and the degree and duration of oxygen desaturation. The SNORAP device is not designed to detect the REM phase of sleep. Moderate and mild OSAS-diagnosed patients (i.e., only occurring during REM), were detected in company with PSG in our laboratory. Our goal is not to determine the REM period via SNORAP. Rather, our aim was to observe polysomnographic changes that occurred, by applying this vehicle, measuring snoring successfully, to the patient groups for whom we already know that attacks of snoring and apnea/hypopnea increase during the REM period. As seen in the findings of our study; although our device did not detect the REM period, it reduced the number and severity of snoring and subsequent apnea attacks. The supine position also contributes to the increase. Oxygen levels that fall overnight during recurrent episodes of apnea-hypopnea (which frequently divides severe snoring and snoring in the supine position), especially in REM sleep, can lead to heart related problems. Heart rate usually slows down during apnea and accelerates after apnea; therefore, arrhythmias can be seen [43]. The SNORAP prototype used in our study was prevented by stimuli sent by respiratory and cardiac events, which seriously increases in the supine position, especially at REM. The heart rate monitor also stimulated a person in situations where the heart rate had risen below 40/min up to 120/min, and helped prevent a possible heart event. SNORAP, which prevented all these events led to the extension of the REM sleep period and therefore REM removed complaints such as “forgetfulness, loss of attention, daytime sleepiness” due to sleep deprivation. Furthermore, complaints of “morning headache and tired wake up” are due to inadequate oxygenation as desaturation overnight.

### 6.5. Time Relationship between Snoring and Apnea

The volume of snoring can vary across patients and across nights for the same patient; it can be continuous or discontinuous. According to acoustic analysis, it may be at a frequency of 200 Hz at the palate level, and of 1000 Hz at the tongue base [6]. It is typical that snoring that occurs in OSAS patients is interrupted by frequent recurrent apnea. Because, although snoring is intermittently interrupted, and air intake and delivery in the mouth and nose stop; abdominal and chest movements paradoxically continue, which has been revealed by our study [44]. Of course, with the SNORAP, the subject of study, we tried to measure real-time snoring volume. Here, we did not intend to measure the time difference between snoring and apnea formation. Besides, we did not also aim to identify NREM and REM phases by the SNORAP device. This is because we observed the recurred apnea/hypopnea attacks of the patients in our study group as polysomnographic, occurring when the patients were in supine position and/or during their REM times during snoring and subsequent periods. Accordingly, SNORAP’s task is to give a tactile stimulus hat will not awaken the individual during the night sleep but causes him to change his sleeping position. Thus, independently of the time difference between snoring and apnea attacks, the severity and number of snoring episodes decrease, and subsequently, the severity and number of apnea/hypopnea episodes decrease.

## 7. Conclusions

As a result of this study, snoring and sleep apnea syndrome were shown to cause harm to both general and sleep health. A group of diseases which can cause damage, but cannot be fully treated have been described, and a device called SNORAP was proposed for the improvement of these diseases. designed by prototyping all of these physiological mechanisms, and demonstrated in the following cases: (a) simple snoring; (b) OSAS with mild and moderate severity; (c) OSAS with REM Dependent Mild and Moderate Degree; (d) OSAS with Mild and Moderate degree; and (e) Patients who received CPAP therapy, but had position-dependent mild and moderate apnea attacks.

Sleep, which accounts for a third of our lives, is a physiological need for our brain and body health. The deterioration of this need, that is, changes in sleeping patterns and duration, diminishes the quality of individual activities during the day. Therefore, the maintenance of sleep health or the correction of the disrupted sleep quality causes the individual and society to lead a higher quality of life.

The most distinguished characteristics of our study, when compared to other studies, is the benefit to certain disease groups as shown by our experimental studies. The second important feature of our study is that snoring sensor has a high success rate. The third important feature of our study is that we are able to monitor heartbeat. Our device increases the intensity and number of vibrating stimuli when the pulse or heart rate drops below 40/min or exceeds 120/min. The fourth important feature of our study is that device is coin-sized, and a device of such size can easily be placed on one of the individual’s anatomical regions with a bandage so as to provide optimal comfort for individual. Our primary goal is to help a special group of patients who we have observed to not get very good help. It has been recommended that a tennis ball be stitched into their night clothes so that patients with the diagnosis of Position-Dependent Mild and Moderate Level OSAS/Snoring can avoid entering the supine position during their night sleep. Moreover, considering patented products with this aim in the world, there is a product designed with belts to hang on the lower back and whose usages are difficult [45]. However, SNORAP is a new, easy to use, minimally invasive (requiring only a wrist or arm band) product giving doctors and patients much more information. At the same time, the AF method described in the method section has been developed. The AF method has been developed in the summarization phase to use less space.

Experimental works on patients at the sleep and electrophysiology lab are still continuing. In order to develop SNORAP, we are trying to integrate an airflow sensor into the system. Thus, in case there is no airflow, SNORAP can recognize it and the patient is warned more effectively by more accurately identifying apnea attacks. At the same time, we want SNORAP to determine REM periods. That is why, our works on EEG are continuing. In particular, we want to ensure in future works that the SNORAP device can determine a PSG sensor’s mission in the home environment.

## Figures and Tables

**Table 1:** Obstructive Sleep Apnea Syndrome Classification [8,9].

AHI	OSAS Degree
<5	Normal
5–15	Mild
16–30	Moderate
>30	Severe

**Table 2:** Patient groups that cannot be proposed CPAP and not fully treated.

(a) Simple Snoring Patients (Position dependent ^1^/REM dependent)
(b) Mild Degree OSAS Diagnosed Patients with Snoring Complaints (Position dependent/REM dependent)
(c) Moderate Severe OSAS Patients with Snoring Complaints (Position dependent/REM dependent)
(d) In normal patient groups (increased snoring and apnea) (Position dependent/REM dependent)

^1^ Snoring in the supine position is exacerbated.

**Table 3:** List of *p* values of statistically significant parameters.

Index Parameters	*p* Value
A/H Index	0.003
Hypopnea Index	0.006
Supine AHI	0.056
REM AHI	0.046

**Table 4:** Method comparison using snoring sounds.

	Accuracy	Method
Wang, C et al. [34]	94%	Sample Entropi + Support Vector Machine
Dafna et al. [35]	98.2%	AdaBoost-based method
Yadollahi [36]	93.2%	Fischer Linear Discriminant + Bayesian
Karunajeewa et al. [37]	96.7%	Zero crossings + energy of the signal + linear predictive coding analysis + noise reduction techniques
Cavusoglu et al. [38]	86.8%	Energy and zero crossing rate
Duckitt et al. [39]	89%	Hidden Markov models + spectrally based features
Our study	98.9%	Audio Fingerprint
